# Genomic Survey of Flavin Monooxygenases in Wild and Cultivated Rice Provides Insight into Evolution and Functional Diversities

**DOI:** 10.3390/ijms24044190

**Published:** 2023-02-20

**Authors:** Yashika Gaba, Bidisha Bhowal, Ashwani Pareek, Sneh Lata Singla-Pareek

**Affiliations:** 1 Plant Stress Biology Group, International Centre for Genetic Engineering and Biotechnology, New Delhi 110067, India; 2 Stress Physiology and Molecular Biology Laboratory, School of Life Sciences, Jawaharlal Nehru University, New Delhi 110067, India

**Keywords:** flavin monooxygenase, abiotic stress, wild rice, auxin biosynthesis, pathogen defense, S-oxygenation

## Abstract

The flavin monooxygenase (FMO) enzyme was discovered in mammalian liver cells that convert a carcinogenic compound, N-N′-dimethylaniline, into a non-carcinogenic compound, N-oxide. Since then, many FMOs have been reported in animal systems for their primary role in the detoxification of xenobiotic compounds. In plants, this family has diverged to perform varied functions like pathogen defense, auxin biosynthesis, and S-oxygenation of compounds. Only a few members of this family, primarily those involved in auxin biosynthesis, have been functionally characterized in plant species. Thus, the present study aims to identify all the members of the FMO family in 10 different wild and cultivated *Oryza* species. Genome-wide analysis of the FMO family in different *Oryza* species reveals that each species has multiple FMO members in its genome and that this family is conserved throughout evolution. Taking clues from its role in pathogen defense and its possible function in ROS scavenging, we have also assessed the involvement of this family in abiotic stresses. A detailed in silico expression analysis of the FMO family in *Oryza sativa* subsp. *japonica* revealed that only a subset of genes responds to different abiotic stresses. This is supported by the experimental validation of a few selected genes using qRT-PCR in stress-sensitive *Oryza sativa* subsp. *indica* and stress-sensitive wild rice *Oryza nivara.* The identification and comprehensive in silico analysis of FMO genes from different *Oryza* species carried out in this study will serve as the foundation for further structural and functional studies of FMO genes in rice as well as other crop types.

## 1. Introduction

The discovery of flavin monooxygenases (FMOs) traces back to the 1960s in mammalian hepatic microsomes, where it was needed to convert N-N′-dimethylaniline into N-oxide [1]). N-N′-dimethylaniline is a tertiary amine compound that has potential carcinogenic activity. Therefore, it requires detoxification by the liver cell [2]. FMOs belong to one of the protein families needed for detoxification of the carcinogens in the cell [3,4,5,6,7,8].

FMOs are found in all domains of life and metabolize several xenobiotic compounds like toxins, several drugs, and pesticides. They need cofactors flavin adenine dinucleotide (FAD), nicotinamide adenine dinucleotide phosphate (NADPH), and dioxygen for their activity [7,9,10,11,12,13]. FMO catalyzes the transfer of hydroxyl groups to small, nucleophilic substrates with heteroatoms like sulfur, nitrogen, iodine, and selenium, thus making them polar and therefore easily excretable from the cell [7,14]. Yeast has a single FMO [15], and animals have five FMOs [16,17,18,19]. The first plant FMO, *YUCCA*, was reported in Arabidopsis in 2001, after almost 40 years of its discovery [20]. YUCCA catalyzes the rate-limiting step in auxin biosynthesis. Previous reports have shown that plants harbour multiple FMO proteins, for example, Arabidopsis has 29 putative FMOs, and cultivated rice, *Oryza sativa* subsp. *Japonica*, has around 20 putative FMOs, thereby indicating that this family might have evolved in plants to perform diverse functions [7,14]

Evolutionary analysis of plant and animal FMOs revealed plant FMOs to belong to three major clades—clade I, clade II and clade III—based on their homology with animal FMOs [21]. Arabidopsis, Poplar, and rice FMOs were then assigned to these clades based on their putative functions like pathogen defence, auxin biosynthesis, and S-oxygenation [7,14,22,23,24,25,26,27]. Several studies have been carried out to identify and/or characterize the FMO genes belonging to the auxin biosynthetic clade (named YUCCAs) in plants [26,27,28,29,30,31,32,33,34,35,36,37,38]. However, only very few reports are available for the other two clades [21,39,40,41,42,43,44]. In addition to the previously reported roles, studies indicate the role of FMOs in abiotic stresses as well. *YUCCA6* overexpression was found to improve drought tolerance and delayed leaf senescence in Arabidopsis plants [45,46]. Furthermore, some wheat FMOs (*TaYUCCA1-A*, *TaYUCCA2-B*, *TaYUCCA3-A*, *TaYUCCA10.1*, *TaYUCCA8-B* and *TaYUCCA10.2*) were involved in heat and drought stress [44]. Despite playing an important role in hormone metabolism, pathogen resistance, signalling, and chemical defence, the identification of FMOs and the novel reactions that they perform, as well as further knowledge of FMO functionality, has remained relatively limited over the last decade and is restricted to a small number of plant species.

Crop wild relatives (CWRs) have the potential to provide a useful pool of genetic material to plant breeders that can help in crop improvement. During the first half of the 20th century, gene pools from wild relatives were used for improvements in sugarcane by introgression. This produced a few disease-resistant “wonder canes” with an increase in cane and sugar yields, improved ratooning ability, and adaptability to various abiotic stresses [47,48,49,50]. In the 1940s and 1950s, CWRs were used in breeding programs for major food crops [51]. Besides trait introgression from wild relatives to cultivated species, research on the genomes of wild relatives has resulted in a process known as neodomestication. Neodomestication is a novel approach for crop improvement that allows the creation of a domesticated crop from a non-domesticated species by manipulating critical domestication genes [52]. The neodomestication of a wild relative of rice, reported in 2021, is the first example of a newly domesticated cereal crop with a polyploid genome. Researchers have used the CRISPR genome editing tool to target the traits controlling seed shattering, plant height, grain size, and flowering time in a wild species, *Oryza alta*, which is salinity stress-tolerant and pest-resistant. As a result, a shorter plant with longer grains and reduced flowering time was developed. These characteristics make *O. alta* suitable for grain production in temperate climates [53,54]. This opens the door to the rapid neodomestication of the wild relatives carrying resilience and adaptation traits for further crop improvement. Thus, the efficient use of genetic diversity from wild relatives would help in new gene discoveries and can be used for crop improvement [55,56,57,58,59,60,61,62].

Rice is of significant value to humans as it is a major staple food for more than half of the world population. The genus *Oryza* has been domesticated independently in Asia (10,000 years ago), which is *Oryza sativa*, and Africa (3000 years ago), which is *Oryza glaberrima.* The global population is expected to increase to 9 billion by 2050. Therefore, rice breeders need to develop new and improved cultivars of rice that have higher yields and adaptation to different biogeographic regions [63,64,65,66]. CWRs of rice can provide genetic resources for these important traits as they are adapted to wide geographic ranges and are tolerant to many biotic and abiotic stresses [63,67,68]. A total of 27 species of the genus *Oryza* are present after 15 million years of evolution, with 11 genome types. Some 6 of them were diploid (*n* = 12, AA, BB, CC, EE, FF, GG) and 5 were polyploid (*n* = 24, BBCC, CCDD, HHJJ, HHKK, KKLL). Cultivated rice is diploid, having an AA genome. For trait introgression from CWRs or their neodomestication, the first step is to gather information about genomic differences and similarities between the wild and cultivated species [52,63]. This can also speed up the discovery of new candidate genes for stress tolerance in plants.

Our lab has been working on identification and characterization of several gene families that contribute to tolerance towards abiotic stresses [64,69,70,71,72,73]. In this study, we have explored the presence of the FMO family of genes in 10 genomes of rice (both wild and cultivated) to understand the evolution, conservation and functional diversification of the FMO family. We have also investigated the possible involvement of this family in providing tolerance to different abiotic stresses using publicly available expression datasets and further experimental validation.

## 2. Results

### 2.1. The Number of FMO Genes Varies in Wild and Cultivated Species of Rice

The hidden Markov model (HMM) profile (PF00743) search was done against the Gramene [74] database for different *Oryza* species (*Oryza brachyantha*, *Oryza punctata*, *Oryza meridionalis*, *Oryza glumaepatula*, *Oryza glaberrima*, *Oryza barthii*, *Oryza nivara*, *Oryza sativa* subsp. *indica*, *Oryza rufipogon* and *Oryza sativa* subsp. *japonica)*. We found that the number of FMO genes, having conserved binding motifs for FAD and NADPH, varies in cultivated and wild rice species. There are 25 different *FMO* members in *O. brachyantha*, 22 in *O. punctata*, 27 in *O. meridionalis*, 30 in *O. glumaepatula*, 24 in *O. glaberrima*, 25 in *O. barthii*, 24 in *O. nivara*, 29 in *O. sativa* subsp. *indica*, 24 in *O. rufipogon* and 28 in *O. sativa* subsp. *japonica*. Table 1 lists the FMO genes found in various species and these species have been arranged according to their evolution. Among these species, *O. brachyantha* is the most distant, while *O. sativa* subsp. *indica* is most recent one. The nomenclature of various genes in different *Oryza* species has been assigned according to their appearance on the chromosomes in ascending order, prefixed by the genus and species name.

### 2.2. The Distribution of FMO Genes on Chromosomes Reveals the Presence of Gene Clusters

**Figure 1:**
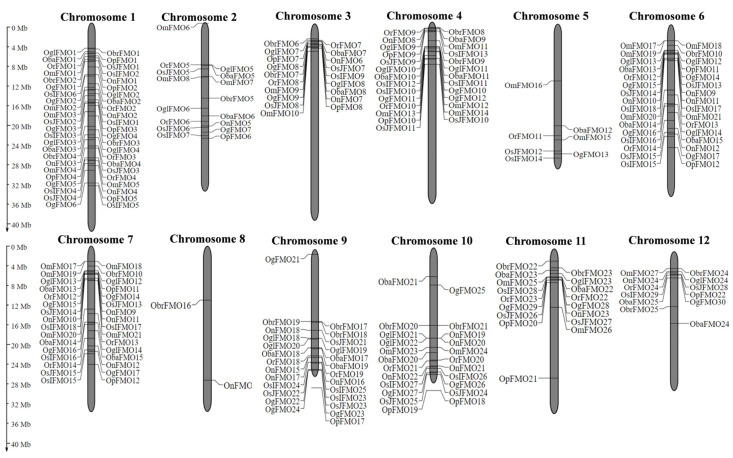
**Chromosomal distribution of FMO genes in wild and cultivated rice.** The distribution of the FMO genes on the twelve chromosomes of rice was determined using MapGene2Chromosome tool. The chromosome numbers are depicted on top of each chromosome. The position of each gene on the respective chromosome has been depicted in terms of megabase pairs by numbers beside each gene.

Information regarding the coordinates of FMO genes belonging to different *Oryza* species was obtained from the Gramene database and genes from all the species were mapped on the rice chromosomes using MapGene2Chromosome v2 [75]. We found that FMO genes are mainly distributed on 11 out of 12 rice chromosomes. Unlike other chromosomes, Chromosome (Chr) number 8 was the least populated, consisting of single FMO gene from *O. brachyantha* (*ObrFMO18*) and *O. nivara* (*OnFMO14*) (Figure 1). Among these 12 chromosomes, the maximum members of a single species are present on Chr 1 and Chr 4 and the lowest are found on Chr 5 and Chr 8. Furthermore, careful observation of chromosomal distribution of FMO genes revealed that the majority of the orthologous FMO genes in different *Oryza* species were found in the same order on a particular chromosome. Also, it is noteworthy that some FMO genes form clusters as they are adjacent to each other on the chromosomes. For example, in *Oryza sativa* subsp. *japonica OsJFMO7* and *OsJFMO8* forms a cluster on Chr 3, *OsJFMO13* and *OsJFMO14* on Chr 6, *OsJFMO16*, *OsJFMO17*, *OsJFMO18*, and *OsJFMO19* on Chr 7, *OsJFMO22* and *OsJFMO23* on Chr 9, *OsJFMO24* and *OsJFMO25* on Chr 10. Further, *OsJFMO26* and *OsJFMO27* forms a cluster on Chr 11. 

### 2.3. Phylogenetic Analysis Shows Conservation among FMO Family and the Majority of Them Belongs to the S-Oxygenating Clade in Different Oryza Species

**Figure 2:**
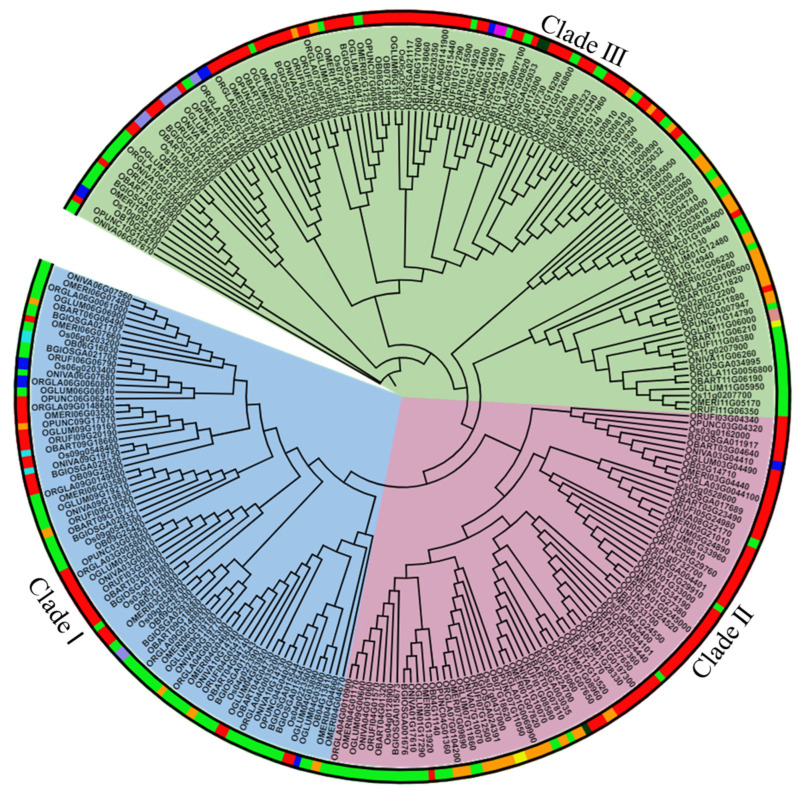
**Evolutionary and sub-cellular localization analysis of FMO genes in wild and cultivated rice.** A rooted circular phylogenetic tree, depicting evolutionary connection between the FMO encoding genes in wild and cultivated rice, was determined by maximum likelihood method with 1000 bootstrap replicates using MEGA 7.0 and visualised using iTOL. The subcellular localization of the genes has been indicated by a color strip around the tree. Red—cytoplasm, parrot green—chloroplast, dark green—endoplasmic reticulum, orange—plastid, blue—nucleus, cyan—vacuole, pink—golgi, peach—cytoskeleton, yellow—extracellular space. The branch lengths indicate the evolutionary time between the two nodes.

To determine the evolutionary and phylogenetic relationships among FMO proteins in different *Oryza* species, a rooted tree was constructed using MEGA7 software [76] and the proteins were grouped into three different clades based on their putative functions (Figure 2). Clade I was assigned as a pathogen defense clade, Clade II as an auxin biosynthesis clade, and Clade III as an S-oxygenating clade based on its constituents’ homology to known and characterized Arabidopsis members [14,21]. Cultivated rice (*O. sativa* subsp. *indica*, *O. sativa* subsp. *japonica*, *O. glaberrima*) has 8 members in Clade I, 6–7 in Clade II and 10–14 members in Clade III. In wild rice species (*O. brachyantha*, *O. punctata*, *O. meridionalis*, *O. glumaepatula*, *O. barthii*, *O. nivara* and *O. rufipogon*), there are 4–9 members in clade I, 5–8 members in clade II and 8–13 members in clade III. Most family members belong to the S-oxygenating clade. We also noticed that OnFMO10 is the outlier in the phylogenetic tree (Figure 2). This might be because of its lower homology to all the other protein sequences of the *Oryza* species.

The localization of FMO proteins was predicted using WolfPsort software [77]. A maximum number of proteins were found to be localized in the, cytoplasm followed by chloroplast (Figure 2). In *Oryza sativa* subsp. *japonica*, 11 proteins were found to be localized in the chloroplast (light green strip), 10 in the cytoplasm (red strip), 4 in the plastid (orange strip), 1 in the nucleus (blue strip), 1 in the peroxisome (purple strip), and 1 in the endoplasmic reticulum (dark green strip). However, in *O. sativa* subsp. *indica*, 13 proteins were found in the chloroplast, 11 in the cytoplasm, 4 in the plastid and 1 in the ER. In wild rice species, besides these organelles, very few FMO proteins were also found to be localized in the vacuole (cyan strip), cytoskeleton (peach strip) and extracellular compartment (yellow strip). Based on the results of multiple sequence alignment by ClustalW and the phylogenetic tree, orthologs from different species were identified. These are represented in Table 2. 

### 2.4. Protein Motif Analysis Reveals Five Conserved Motifs in FMO Members from Different Oryza Species

**Figure 3:**
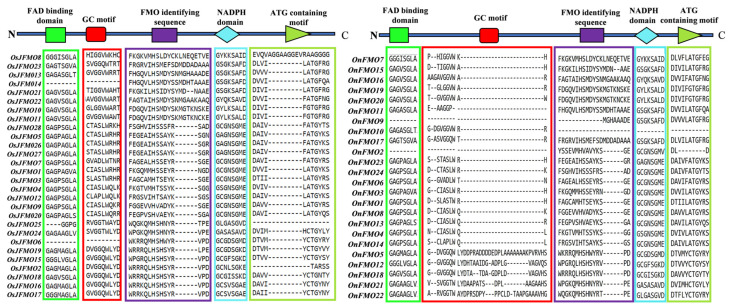
**Schematic representation of the conserved FMO-identifying motifs in the FMO proteins in wild and cultivated rice.** FMO-identifying motifs previously reported in literature were searched for in the FMO protein sequences of the cultivated *Oryza sativa* subsp. *japonica* and wild species, *Oryza nivara.* The motif sequences have been marked by colours representing each of the conserved motifs, bright green: FAD binding domain; red: GC motif, violet: FMO-identifying sequence; cyan: NADPH domain and light olive green: ATG-containing motif.

Following the phylogenetic analysis of FMO members in different *Oryza* species, we analyzed the conservation of different motifs, 5 of which are signatory conserved motifs of FMOs. It has been reported that FMO binds the adenine motif of FAD with a conserved GxGxxG sequence which is the FAD-binding motif found at the N terminus of the protein [78]. An identical (GxGxxG) but less conserved motif, the NADPH-binding motif is located at the centre of the protein [79]. Another motif, ATG containing motif (ATGY) is present at the C-terminus and occurs in proteins that carry out N-oxidation. Finally, a conserved motif FxGxxHxxY/F, FMO-identifying sequence is found in all known plant FMOs. It can be distributed anywhere in the protein [79]. We found that orthologs from different *Oryza* species exhibit high conservation in protein motifs (Figure 3). Variation in protein motif conservation in some orthologs from *Oryza brachyantha* has been observed as it is the most distant ancestor among these species. We also checked the presence of signatory conserved motifs of the FMO family. 

**Figure 4:**
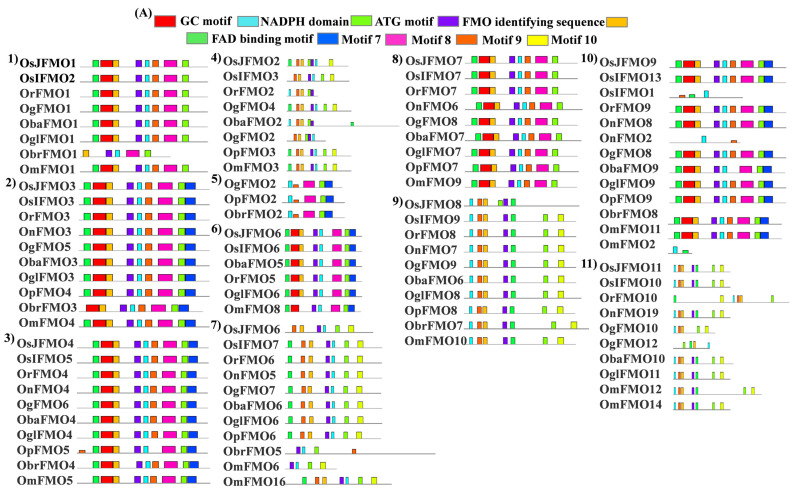

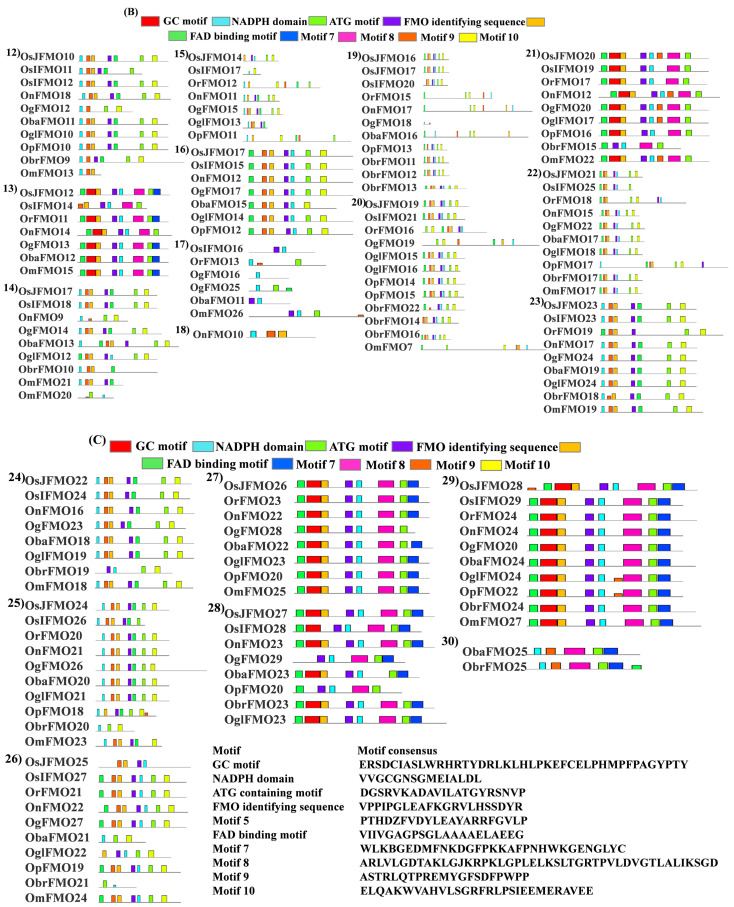
**Protein motif analysis in FMO genes in wild and cultivated rice.** Conserved motifs, in addition to the FMO-identifying motifs, were analyzed using the MEME suite. The sequences of all the analyzed motifs have been depicted in the box. The FMO protein motifs in the wild and cultivated rice have been represented in sets (**A**) 1–11 (**B**) 12–23 and (**C**) 24–30. Wild rice FMO orthologs have been grouped together in each set. Similar motif organization was observed in the different sets.

In our analysis, we found that almost all of these motifs were present in the protein sequences of different *Oryza* species. However, few of them lack these motifs. The FAD-binding motif was mostly present at the N terminus of the protein, while the ATG-containing motif was present at the C terminus. Also, it was observed that ATG-binding motif was present twice in some of the proteins. Besides these, 5 more conserved motifs were seen in these sequences, which are named motif 5, motif 7, motif 8, motif 9, and motif 10 in this study. Again, OnFMO10 (Figure 4B(18)) shows the difference in protein motif arrangement from the rest of the members, with only 3 motifs present in total, NADPH-containing motif, motif 5, and motif 9. OgFMO16 (Figure 4B(17)) shows the presence of only 1 motif, the NADPH-binding motif, and OsIFMO16 shows the presence of two motifs, NADPH and FMO-identifying motifs (Figure 4A,B). This is probably because this gene has not been sequenced properly and the available sequence in the Gramene database has missing 5′ bases, which are denoted as a stretch of N in the database. In the 5th and 30th sets (Figure 4A(5),C(30) of orthologs that have only wild rice members, the FMO-identifying sequence was absent while 5–6 other conserved motifs were present. 

### 2.5. Analysis of Gene Architecture Depicts Conservation among Orthologs from Different Oryza Species

**Figure 5:**
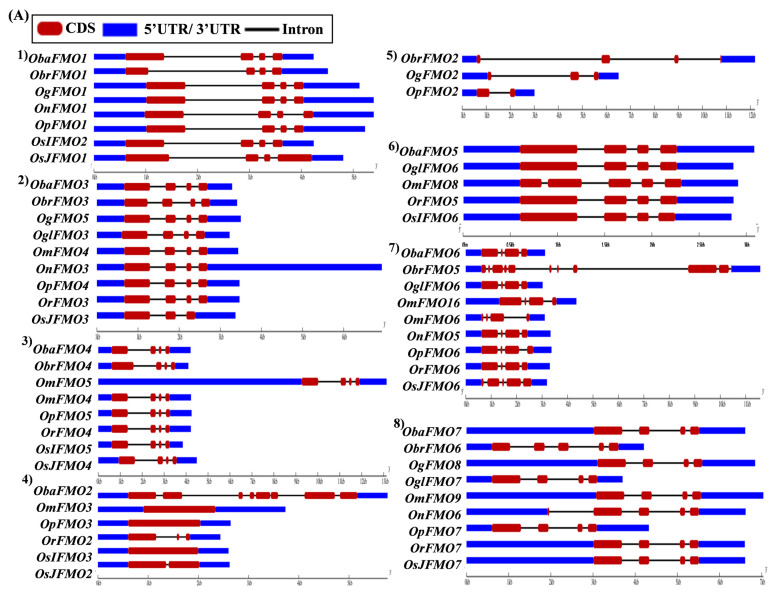

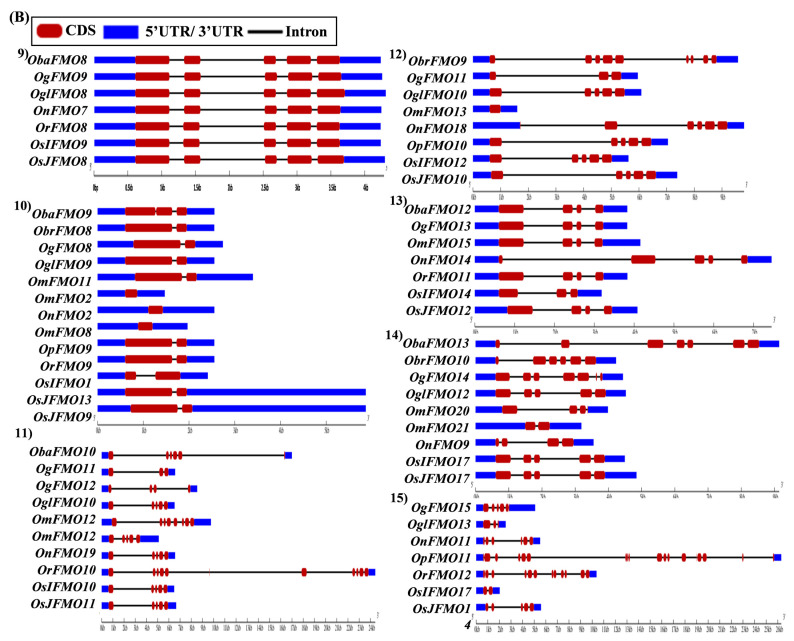

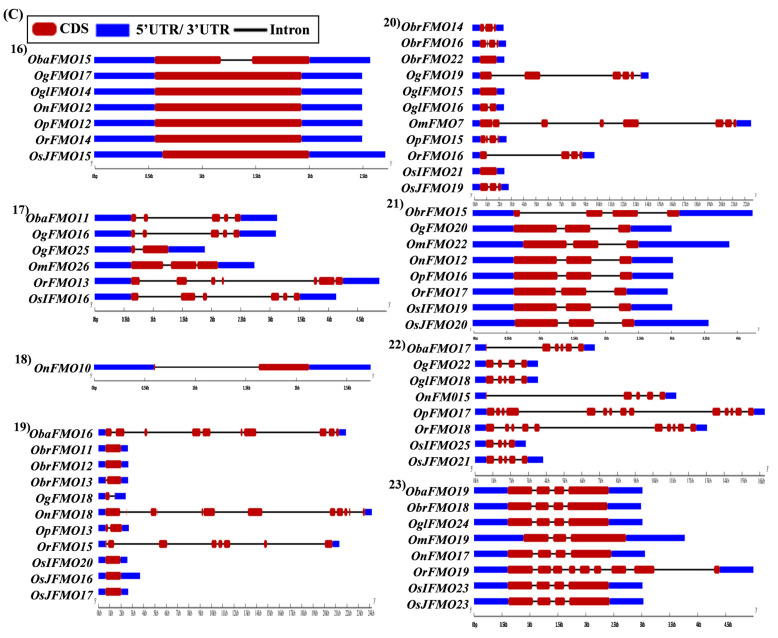

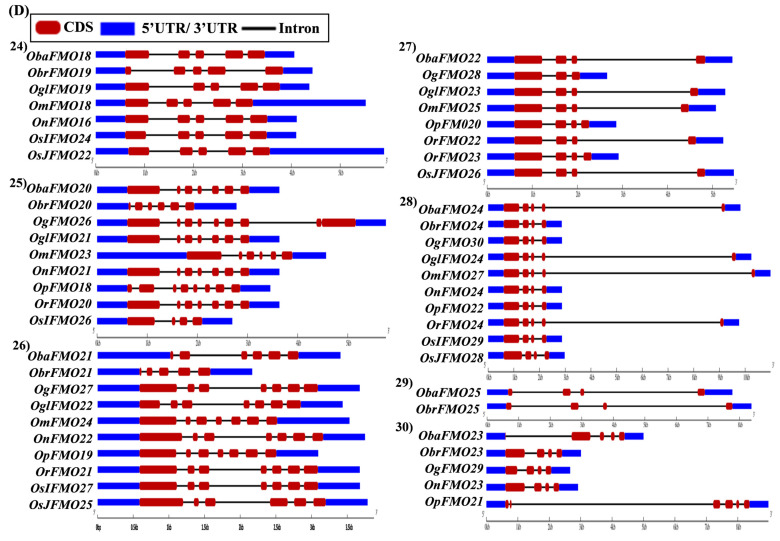
**Gene architecture of the FMO gene family in cultivated and wild relatives of rice.** The exon–intron arrangement of the FMO gene family from the different *Oryza* species, (**A**) 1–8, (**B**) 9–15, (**C**) 16–23 and (**D**) 24–30 was predicted using the Gene Structure Display Server 2.0. The length of UTR, exon, and intron has been depicted in proportion to the actual sizes, which is also indicated using a scale at the top, blue: 5′ UTR/3′ UTR region; red: CDS; solid line: intron. Different sets show conserved gene structures.

The exon–intron arrangement for the genes encoding the FMO proteins in different *Oryza* species was evaluated using the Gene Structure Display Server tool [80]. The gene structures of the orthologous genes have been represented together in Figure 5. The orthologs were largely found to share similar exon–intron arrangements, with a few exceptions. *OnFMO3* possessed an exceptionally long 3′ UTR, while *OmFMO5* had a long 5′ UTR (Figure 5A(2,3)). The exon–intron arrangement of *ObaFMO2* was found to be completely different from that of the other members in the set, consisting of eight exons, while the rest comprised one to a maximum of three exons (Figure 5A(4)). In the fifth set, *ObrFMO2* had longer introns as compared to *OgFMO2* and *OpFMO2* (Figure 5A(5)) A similar observation was also made for *ObrFMO5*, *ObaFMO10*, *OpFMO11 and OrFMO15* and (Figure 5A(7),B(11,15),C(19)).

In the nineteenth set, *ObaFMO16*, *OnFMO18*, and *OrFMO15* consisted of 8–10 exons, while the rest were either intronless or consisted of a maximum of two exons. Another example of this arrangement would be the twentieth set, wherein *OgFMO19*, *OmFMO7* and *OrFMO16* contained varying numbers of exons ranging from 4–8, while others consisted of two exons or no introns at all (Figure 5C(19,20)). In the sixteenth set, all the genes were intronless except *ObaFMO15* (Figure 5C(16)). In the twenty-eight set of orthologs, *ObaFMO24*, *OmFMO27*, *OrFMO24* and *OglFMO24* consisted of longer introns and five exons, one more than the other genes in the same set (Figure 5D(28)). Overall, it can be seen that the *FMO* gene family has variable sizes of exon, intron, and UTRs.

### 2.6. Domain Assessment Reveals Loss of Extra Copies of FAD Domain during the Course of Domestication

**Figure 6:**
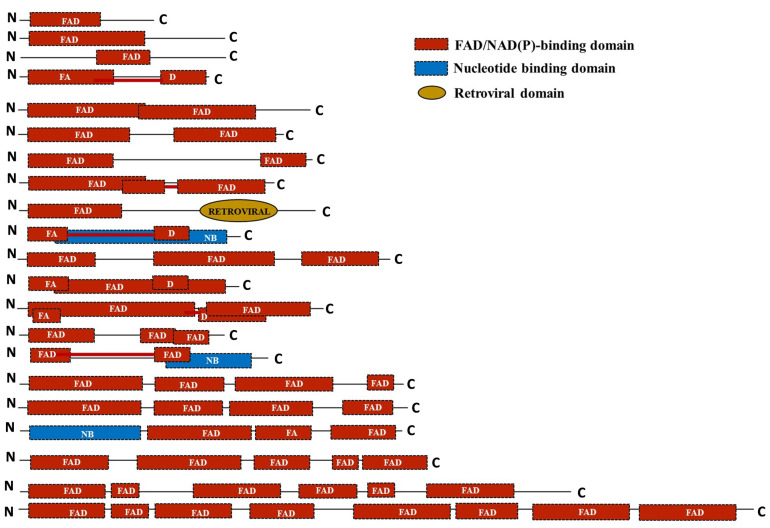
Schematic representation of the domain architecture of the FMO family of proteins in the cultivated and wild relatives of rice. The domain organization of the FMO members in different *Oryza* species was predicted by the SUPERFAMILY database. FAD; flavin adenine dinucleotide domain, NB; nucleotide-binding domain; retroviral domain. The length of the domains has been represented in proportion to their actual sizes and are not to scale. Domains connected by a red line mean they are split.

To assess the domain architecture of the identified putative FMO proteins, analysis was done using the SUPERFAMILY database [81]. The protein sequences were found to harbour single-to-multiple copies of the FAD/NADP-binding domain. Additionally, a nucleotide-binding domain and retroviral domains were also found in a few of the sequences like OgFMO19, OgFMO2, ObrFMO25, and ObaFMO2, respectively. While all the FMO family proteins of the cultivated rice varieties, both indica and japonica, consisted of single-to-double repeats of the domain, wild relatives of rice mostly contained two-to-five repeats of the domain such as OpFMO11, OpFMO17, OrFMO15, OrFMO18, OnFMO18, OgFMO19. OnFMO18, and OgFMO19. In fact, OmFMO7 was found to harbor eight copies of the domain, the most among all the proteins in wild vs cultivated varieties. The presence of multiple domains also correlated with the sequence length of the proteins. Interestingly, we also found a few of the domains to be arranged in such a way that they overlapped with the adjacent domain repeat, while others were split because they were linked together by a linker or spacer region (Figure 6). 

### 2.7. Developmental and Stress-Mediated Expression Profiling of FMO Genes Reveals Tissue Specificity and Perturbations in a Subset of Genes in Different Abiotic Stresses

Next, we analyzed the expression profiling of the genes encoding the FMO family of proteins in the cultivated variety *Oryza sativa* subsp. *japonica*. Normalized and curated transcript abundance data were retrieved from the publicly available Genevestigator database. Of the 28 genes, expression data for 6 genes viz, *OsJFMO2* (Os01g0368000), *OsJFMO12* (Os05g0528600), *OsJFMO17* (Os07g0111900), *OsJFMO18* (Os07g0112000), *OsJFMO26* (Os11g0207700), *OsJFMO27* (Os11g0207900) could not be retrieved due to data unavailability. 

**Figure 7:**
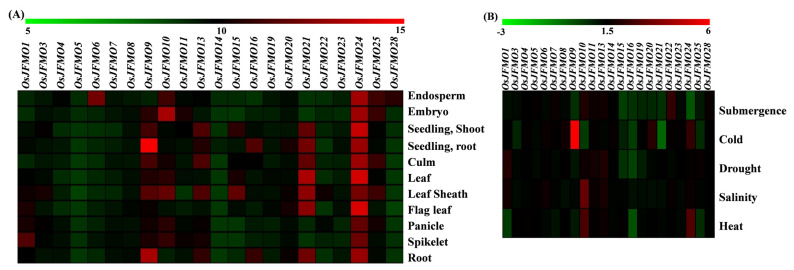
**Spatial and stress-mediated expression profiling of different FMO genes cultivated variety *O. sativa* subsp. *japonica***. Expression data were retrieved from the publicly available Affymetrix as well as RNA-seq datasets from the Genevestigator for (**A**) different tissues during development and (**B**) under different abiotic stress conditions namely heat, salinity, cold, drought and submergence. All expression values with *p* value < 0.05 have been depicted in log_2_ fold change. The heatmaps have been generated using MeV software. The color scale below each heatmap shows the level of expression, with red color showing the highest expression and green showing the lowest expression.

The spatial expression profiling revealed differential expression of the genes in the different tissues, namely endosperm, embryo, seedling, culm, leaf, flag leaf, panicle, spikelet, and root. *OsJFMO6* and *OsJFMO28* were found to be highly expressed only in the endosperm. Most of the other genes such as *OsJFMO5*, *OsJFMO8*, *OsJFMO14*, *OsJFMO19*, *OsJFMO22*, *OsJFMO23*, *OsJFMO3*, *OsJFMO4*, *OsJFMO20*, and *OsJFMO7* showed low to medium level of expression in all the tissues. Interestingly, the transcript abundance of only one gene, *OsJFMO24*, was maintained across tissues. *OsJFMO21* and *OsJFMO9* showed medium-to-high levels of expression in all the tissues. In fact, *OsFMO9* showed maximum expression (14.36-fold change) in roots as compared to other genes. *OsJFMO9* and *OsJFMO10* were found to be highly expressed in the panicle (11.66- and 10.82-fold, respectively) and spikelet (11.95- and 11-fold, respectively). *OsJFMO9* was highly induced (13.33-fold change) in the roots of the seedling. *OsJFMO25* and *OsJFMO9* showed higher expression (11.26-, 11.04-, and 10.79-fold) in the embryo. (Figure 7A).

Further, we explored the expression profiling of the FMO genes under various stress conditions such as salinity, heat, cold, drought, and submergence. All expression values have been denoted as log2 fold change. Out of 28, 10 genes show little or no perturbations under various stresses examined. These are *OsJFMO4*, *OsJFMO5*, *OsJFMO6*, *OsJFMO7*, *OsJFMO8*, *OsJFMO11*, *OsJFMO13*, *OsJFMO14*, *OsJFMO23*, and *OsJFMO28*. However, 11 genes, *OsJFMO1*, *OsJFMO3*, *OsJFMO9*, *OsJFMO10*, *OsJFMO15*, *OsJFMO16*, *OsJFMO19*, *OsJFMO20*, *OsJFMO21*, *OsJFMO24*, and *OsJFMO25* were found to be significantly repressed in one or the other stress. Only a few of them were found to be upregulated. For example, *OsJFMO10* upregulation was highly induced under salinity (2.44-fold) stress. *OsJFMO1* is the only gene found to be upregulated under drought stress. *OsJFMO9* was found to be 5–6-fold upregulated under cold stress, the highest level among all the genes (Figure 7B). Overall, it seems that stresses impose perturbation in a subset of genes among this family. The expression data of each gene has been added in the Supplementary Materials (Table S1).

**Figure 8:**
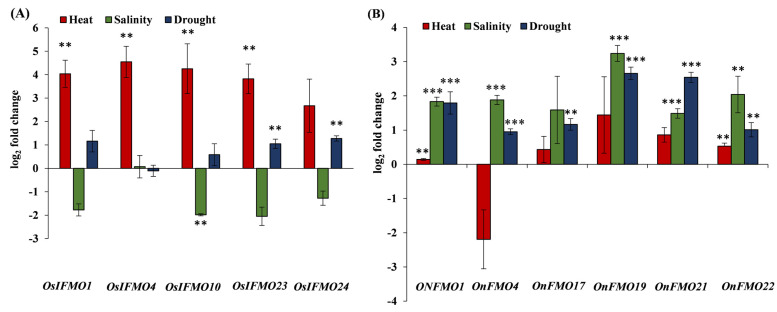
**Real-Time expression analysis of FMO genes in wild and cultivated rice**. Expression profiling of selected FMO members from one month old leaves of (**A**) *Oryza sativa* subsp. *indica* (PB1 variety) and (**B**) *Oryza* nivara using qRT-PCR in response to heat stress (42 °C), salinity stress (200 mM NaCl) and drought stress (water withheld) for 24 h. Mean fold change (log_2_) is depicted, and the expression data is plotted against the untreated samples. The error bar represents the standard deviation where *n* = 6. *** signifies *p* value < 0.05 upto four or more decimal places and ** signifies *p* value < 0.05 for two decimal places.

Next, we validated some of the genes encoding for the FMO family of proteins based on the publicly available stress-mediated expression profiling data. The genes selected belong to the three different clades. Leaves cut from one month old plants of the cultivated species *Oryza sativa* subsp. *indica* and its closest wild rice relative, *Oryza nivara*, were subjected to different stress conditions such as high temperature, salinity, and drought. In *Oryza sativa* subsp. *indica*, *OsIFMO1*, *OsIFMO4*, *OsIFMO10*, *OsIFMO23* and *OsIFMO24*, five genes were found to be significantly upregulated under high temperature stress. However, none of the genes show significant upregulation under drought stress. A contrasting trend was observed in salinity stress where *OsIFMO1*, *OsIFMO10*, *OsIFMO24* and *OsIFMO10* shows downregulation (0.07 to −2.04-fold) (Figure 8A). Under drought stress, all of the genes, *OnFMO1*, *OnFMO17*, *OnFMO21*, *OnFMO4*, and *OnFMO20*, were significantly up regulated in the wild relative *O. nivara*. In heat stress, four of them, *OnFMO1*, *OnFMO17*, *OnFMO19*, *OnFMO21* and *OnFMO22* shows upregulation. In salinity stress, *OnFMO19* (log 2-fold change, 3.24) and *OnFMO22* (log 2-fold change, 2.04) shows perturbation in a significant range (Figure 8B). The ortholog of *OnFMO22* i.e., *OsIFMO27*, was not detected in any of the stresses, thus indicating null expression of the gene at this developmental stage. This is the first report on experimentally validated expression profiling of FMO encoding genes from the wild rice *O. nivara*. Our expression profiling data was mostly, but not entirely, correlated with publicly available expression data of *Oryza sativa* subsp. *japonica*. *OsIFMO10* and *OsIFMO24* show a similar trend in heat and drought stress but an opposite trend in salt stress. Similarly, *OsIFMO1* and *OsIFMO4* show similar trends in drought and salinity, but opposite trends in heat stress. Thus, it can be concluded that, while *FMO* encoding genes in indica variety are induced by heat, their orthologs in *O. nivara* are induced by salinity and drought.

## 3. Discussion

Plant FMOs are currently a significantly under-utilized class of enzymes, but studies in animal systems show that they have a rich potential for the discovery of new activities and novel bioactive metabolites. The evolutionary diversity of plant FMOs can provide a remarkable untapped resource of “green” biocatalysts [7]. Plant FMOs could provide unique breeding targets for subsistence agriculture based on their known important functions in plant metabolism. The identification of the entire complement of FMOs from *Oryza* species used in this study is a significant step forward in FMO research. The comparison of FMO genes from different *Oryza* species also provides useful information on evolutionary aspects of this gene family and shows conservation which clearly indicates the mandatory roles of FMOs, which cell cannot afford to change. However, all the substrates and reactions catalyzed by FMOs are yet to be identified. 

The difference in the number of FMO genes in different *Oryza* species indicates the gain or loss of FMOs during the evolution of individual species. The size of gene families can increase by single gene duplications or duplication of a segment of chromosome i.e., segmental duplication. Gene family size can reduce by single gene deletion or deletion within a segmental piece [82,83]. The most distant ancestor among these species, *O. brachyantha*, has 25 FMO members, *O. punctata* has 22, and cultivated species *O. sativa* subsp. *indica* and *O. sativa* subsp. *japonica* have 28 and 29 genes, respectively. This increase in number in domesticated rice clearly indicates gene duplication events in their genome. There are a few non-functional FMOs in animal systems, such as human *FMO6*, which is a pseudogene [19,84]. Pseudogenes have missing promoters/deleted sequences/%frameshifts/a lesser number of introns or premature stop codons [85,86]. It is possible that a few of them are non-functional in rice as well. Across different *Oryza species*, a variable number of gene members of the jumonji C domain-containing protein family and the CBS-domain-containing protein family have been observed, implying that change in gene family size is very common in evolution [64,87,88]. 

Tandem duplications, which are duplication of adjacent identical chromosome segments (exon(s)/gene) resulting in the formation of another gene or specific exon, occur frequently in the plant genome [89]. The presence of *FMO* gene clusters on different chromosomes might be a result of tandem duplication. Similar clusters were also observed in FMOs belonging to clade II in wheat [90]. The Arabidopsis receptor-like kinase (RLKs) family has been expanded via tandem duplication [91]. Furthermore, it has been reported that tandem duplication has resulted in genome size expansion in rice, which may have occurred specifically in the rice lineage as a response to different stresses [92]. These duplications within the gene families may benefit the species in terms of functional diversity and response. Also, the majority of the orthologous FMO gene family members are found on the same order on chromosomes, indicating that these orthologous genes are present as syntenic blocks in chromosomal regions. 

Phylogenetic analysis reveals that the gene family is conserved across these species due to sequence similarity. Also, the presence of maximum FMO members in clade III indicates their role in S-oxygenation. In 2007, the first plant FMO belonging to this clade was isolated and characterised from Arabidopsis [21]. FMO_GS-OX_ is one out of seven FMOs present in Arabidopsis from this clade [41]. This FMO catalyzes the S-oxygenation of methionine-derived glucosinolates (GSLs) like methylthioalkyl. GSLs are plant secondary metabolites that are present in cruciferous plants, especially brassicales. GSLs and their hydrolytic products are involved in various plant functions like defense against pathogens and herbivores [93]. FMOs belonging to rice and other plant species that do not produce GSLs, on the other hand, may be involved in the S-oxygenation of a wide range of sulfur-containing compounds which are widespread in the plant kingdom [39]. However, the actual substrates of these FMOs are not known. The localization of FMOs in different intracellular compartments might provide functional diversity to this gene family by simultaneous physical segregation and the operation of various metabolic processes in the same cell.

The majority of FMO proteins from these species have conserved motifs that have been reported in the literature. The absence of some conserved motifs in a few FMO members again raises the possibility that these are non-functional pseudogenes but are related to other members of this family due to significant sequence similarity. Based on the absence of few conserved motifs, recent identification of NRL [NONPHOTOTROPIC HYPOCOTYL 3/ROOT PHOTOTROPISM 2-like (NPH3/RPT2-Like)] genes from rice have revealed the presence of two pseudogenes among the 27 that are present in the genome [94]. In fact, rice has many pseudogenes that belongs to different gene families [95]. However, the presence of these non-functional proteins in the FMO family can only be confirmed through functional studies.

Most orthologs from different *Oryza* species have similar exon–intron arrangements, again demonstrating the conservation of this gene family. However, the presence of an exceptionally long 3′ UTR in *OnFMO3* and 5′ UTR in *OmFMO5* indicate its role in specific developmental processes, as longer 5′ UTRs are generally found in genes involved in regulating growth processes in a tissue-specific manner [96]. The FMO-encoding genes with longer introns may be highly expressed as compared to those with shorter or no introns since, in plants, generally, genes with longer intronic regions and UTRs have high transcript abundance [97,98,99]. Also, it has been shown that the presence of both longer 3′ UTRs and introns act as cis elements to promote non-sense mediated decay (NMD) which is a quality check mechanism in eukaryotes including plants to eliminate identified aberrant mRNAs with a premature termination codon [100]. Thus, it is possible that *OnFMO3*, *OmFMO5*, and *ObrFMO2* may be subjected to NMD owing to the presence of longer UTRs and introns in its gene architecture. Different gene structures in some sets of orthologs also indicate that gene duplication in this family has been followed by subsequent diversification [101,102] which can help these FMOs in performing various functions and/or in spatio-temporal regulation. The variable sizes of exon, intron, and UTRs in the *FMO* gene family implies that genes have undergone extensive shuffling during evolution [103,104]. 

From domain analysis, we have found that wild rice strains have mostly 2–5 FAD domains, with few members having more than five domains as well, whereas cultivated rice strains have only 1–2 FAD domains. There are three possibilities here. First, all the FAD domains in wild rice species work equally, and to reduce the genetic load on the cell, some of them have been deleted and the complete function has been assigned to a lesser number of domains in the cultivated rice. Second, only 1 or 2 domains are actually functional and are sufficient for the function of an FMO, and so the extra copies of this domain have been deleted. Another possibility is that these domains may be split in wild rice and together, they make functional domains during protein folding. A recent study has shown that domain gains and losses occur frequently during proteome evolution and concurrently with the evolution of cells. Fascinatingly, the number of gains tends to exceed the losses in the proteome, which could be to redefine the survival strategy of an organism [105]. In fact, it has been shown that higher eukaryotes trade-off the organismal budget for possessing the unique number of genes and domain architecture (economy), with the ability to resist damage and adaptability to environmental change [106]. It seems possible that a sharp reduction in the genetic size, together with a tendency to retain flexibility and robustness during domestication, has resulted in the subsequent loss of the extra repeats of the FAD domain of the FMO family in the cultivated varieties. Furthermore, the presence of nucleotide-binding domains in some FMO proteins clearly indicates that some members of this family play a role in pathogen defense, as this domain aids in pathogen recognition and signalling [107,108,109,110].

From the in silico expression analysis of available FMO genes of *O. sativa* subsp. *japonica* in different tissues, few genes were found to be highly expressed in specific tissues such as endosperm, root, panicle, spikelet, seedling root and embryo. Expression in a particular tissue indicates their functional specificity. The majority of the genes’ expression, however, was consistent across all tissues. Therefore, it can be inferred that these genes might be playing a significant role across all the developmental processes. Expression analysis of FMO genes under different abiotic stresses, as well as validation by qRT-PCR, reveals that the entire family does not respond to these stresses where only a subset of genes get downregulated and some were upregulated under these stresses. Some of the FMO genes belonging to Clade II have been found to be downregulated in drought and heat stress in wheat also [90]. In our study, we also found that few genes are downregulated under salinity stress, thus indicating the possibility that some of the members of the *FMO* family are not needed in these stresses and probably that its silencing could play a significant role in imparting stress tolerance in rice like *SlMYB50* and *SlMYB55* genes, the silencing of which enhanced salinity and drought tolerance in tomato [111,112]. However, this data is very preliminary, and detailed spatio-temporal and multiple stress-mediated expression profiling would help us understand the trend better. We believe the findings of this study will aid in the functional characterization of this gene family in rice, opening up new avenues for future research.

## 4. Materials and Methods

### 4.1. Identification of the FMO Family Members in Rice

The HMM (hidden Markov model) search of the FMO domain (PF00743) was retrieved from the Pfam database, (http://pfam.xfam.org/; EMBL-EBI, UK; accessed on 12 July 2022), which was used to identify the full-length protein sequences of FMO in the Gramene [74] database (https://www.gramene.org, accessed on 12 July 2022) for the identification of homologous sequences in 10 Oryza species: *Oryza sativa* subsp. *japonica*, *O. sativa* subsp. *indica*, *O. barthi*, *O. brachyantha*, *O. glumaepatula*, *O. glaberrima*, *O. meridionalis*, *O. nivara*, *O. punctata*, and *O. rufipogon*. All the retrieved sequences were analyzed using the Pfam 34.0 database (http://pfam.xfam.org/, accessed on 13 July 2022) for the presence of FAD domain in the FMO gene family. The developers and publishers of the Pfam database have chosen to shut down the website and reroute traffic to (InterPro (ebi.ac.uk)), another web-based resource that, like Pfam, analyses protein sequences functionally by grouping them into families and predicting the presence of domains and important sites. The data from the two databases are correlated.

### 4.2. Phylogenetic Analysis

Multiple sequence alignment of the FMO proteins from different *Oryza* species was performed using Clustal Ω and subsequently, phylogenetic tree was constructed using MEGA 7.0 [76] by the maximum likelihood method with 1000 bootstrap replicates. Tree was visualized using iTOL (https://itol.embl.de/upload.cgi, accessed on 14 July 2022). For subcellular localization, protein sequences were analyzed using the Wolf Psort: Protein subcellular localization predictor (https://wolfpsort.hgc.jp/, accessed on 12 July 2022).

### 4.3. Motif Analysis

The identified FMO sequences were analyzed for the presence of conserved motifs using the multiple expectation maximization for motif elicitation (MEME) program (http://meme-suite.org/, accessed on 27 July 2022), with the default parameters and the maximum number of motifs set to 10.

### 4.4. Evaluation of Domain Architecture of the FMO Family Proteins

The proteins sequences of the genes encoding the FMO family of proteins were analyzed using the SUPERFAMILY database (SUPERFAMILY database of structural and functional protein annotations for all completely sequenced organisms (supfam.org), accessed on 28 July 2022) for SCOP assessment using the default parameters. 

### 4.5. Evaluation of the Gene Structure

The architecture of the genes encoding FMO proteins was determined and visualized using the Gene Structure Display Server tool, using genome sequence and CDS as query sequence (Gene Structure Display Server 2.0 (gao-lab.org), accessed on 29 July 2022).

### 4.6. Developmental and Stress-Mediated Expression Profiling of the FMO Encoding Genes

Normalized curated expression profiling data of the FMO encoding genes under different stresses and in the various anatomical parts under different developmental stages were retrieved from the Affymetrix, as well as RNA-seq datasets of the publicly available Genevestigator database (https://genevestigator.com; NEBION, Switzerland; accessed on 4 August 2022). The heatmaps were generated using MeV 4.9.0 application tool.

For qRT-PCR-based expression profiling of selected FMO genes, leaves cut from one month old plants of *Oryza sativa* subsp. *indica* and its closest wild relative *Oryza nivara*, growing under controlled greenhouse conditions, were subjected to salt stress (200 mM NaCl), drought (water withheld) and high temperature (42 °C) for 24 h. Total RNA was isolated using Trizol reagent (ThermoFisher Scientific, Waltham, MA, USA) and first-strand cDNA synthesis done using the RevertAid first-strand cDNA synthesis kit (ThermoFisher Scientific, Waltham, MA, USA). Real-time PCR was performed to the Applied Biosystems 7500 Step-One Instrument (Applied Biosystems 7500, Foster City, CA, USA). The eukaryotic *Elongation Factor-1α* (*eEF-1α*) was used as an internal control for normalization in the qRT-PCR. The log_2_ expression values of each gene under salinity, heat and drought conditions have been calculated with respect to the untreated control (having log2 value 0) [113].

## 5. Conclusions

Rice genome sequencing has substantially sped up the discovery and characterization of breeder’s important genes, as well as a new understanding of their evolutionary history. In our study, we have identified the entire complement of the FMO family from 10 different *Oryza* species including both wild and cultivated. Wild and cultivated rice species have different numbers of FMO genes. Phylogenetic, motif, and gene structure analysis indicate that this family is conserved across these species. The phylogenetic analysis of FMO orthologues in cultivated rice and its wild relatives also reveals that nearly all of the orthologs that shared comparable structural organizations were clustered together with a convincing bootstrap value (1000). Some of the genes from *O. brachyantha* show a difference in motif and gene structure from other orthologs because it has FF genome and it is the most distant ancestor. Unlike, *O. brachyantha*, *O. punctata* which has the BB genome, is different from the rest of the *Oryza* species with an AA genome and did not show any difference among its orthologs. From domain analysis, we have found that wild rice species have multiple domains which have been subsequently lost during evolution of our cultivated species. Also, the expression of this gene family in different rice tissues is reported in this study. From the in silico expression analysis and validation of selected genes by qRT-PCR in cultivated rice, *O. sativa* subsp. *indica* and wild rice *O. nivara* under different abiotic stresses, it can be concluded that the entire family does not respond to different stresses, implying that they perform diverse functions in the cell. Altogether, this study provides the total number of FMO genes present in rice, which has not been reported till now, and also assesses the similarities and differences in this family across different *Oryza* species. This information can help researchers in the functional characterization of rice FMOs. The stress responsiveness of some FMOs reported in this study further encourages the researcher to use them for improving stress tolerance in the cultivated rice varieties or neodomestication of CWRs. 

## Figures and Tables

**Table 1:** **Identification and nomenclature of FMO encoding genes in wild and cultivated rice.** The names of the FMO encoding genes identified, from the Ensembl Gramene database, have been assigned according to increasing order of chromosomal distribution. Suffixes of OsI, OsJ, Osgl have been apportioned to the cultivated varieties indica, japonica and glaberrima respectively and Oba, Obr, On, Om, Or and Op have been allocated to *O. barthii*, *O. brachyantha*, *O. nivara*, *O. meridionalis*, *O. rufipogon and O. punctata*, respectively. (A) shows nomenclature for *O. brachyantha*, *O. punctata*, *O. meridionalis*, *O. glumaepatula*, *O. glaberrima* and (B) show nomenclature *for O. barthii*, *O. nivara*, *O. indica*, *O. rufipogon* and *O. japonica*. In the table, each rice chromosome is indicated by a different colored row.

**(A) Chromosome**	***O. brachyantha***	***O. punctata***	***O. meridionalis***	***O. glumaepatula***	***O. glaberrima***
1	ObrFMO1 (OB01G18060)	OpFMO1 (OPUNC01G07650)	OmFMO1(OMERI01G07820)	OgFMO1 (OGLUM01G08960)	OglFMO1 (ORGLA01G0069900)
	ObrFMO2 (OB01G21130)	OpFMO2 (OPUNC01G10840)	OmFMO2 (OMERI01G13920)	OgFMO2 (OGLUM01G12480)	OglFMO2 (ORGLA01G0126800)
	ObrFMO3 (OB01G33700)	OpFMO3 (OPUNC01G16290)	OmFMO3 (OMERI01G14340)	OgFMO3 (OGLUM01G17290)	OglFMO3 (ORGLA01G0197300)
	ObrFMO4 (OB01G38810)	OpFMO4 (OPUNC01G24520)	OmFMO4 (OMERI01G24550)	OgFMO4 (OGLUM01G17860)	OglFMO4 (ORGLA01G0245000)
		OpFMO5 (OPUNC01G29760)	OmFMO5 (OMERI01G27380)	OgFMO5 (OGLUM01G28330)	
				OgFMO6 (OGLUM01G33960)	
2	ObrFMO5 (OB02G29400)	OpFMO6 (OPUNC02G19680)	OmFMO6 (OMERI02G00100)	OgFMO7 (OGLUM02G22140)	OglFMO5 (ORGLA02G0106500)
			OmFMO7 (OMERI02G12510)		OglFMO6 (ORGLA02G0187600)
			OmFMO8 (OMERI02G12660)		
3	ObrFMO6 (OB03G14710)	OpFMO7 (OPUNC03G04320)	OmFMO9 (OMERI03G04440)	OgFMO8 (OGLUM03G04490)	OglFMO7 (ORGLA03G0044100)
	ObrFMO7 (OB03G16080)	OpFMO8 (OPUNC03G05820)	OmFMO10 (OMERI03G05840)	OgFMO9 (OGLUM03G06010)	OglFMO8 (ORGLA03G0058000)
4	ObrFMO8 (OB04G11140)	OpFMO9 (OPUNC04G01360)	OmFMO11 (OMERI04G01170)	OgFMO10 (OGLUM04G03890)	OglFMO9 (ORGLA04G0010900
	ObrFMO9 (OB04G13610)	OpFMO10 (OPUNC04G04050)	OmFMO12 (OMERI04G04660)	OgFMO11 (OGLUM04G03900)	OglFMO10 (ORGLA04G0035200)
			OmFMO13 (OMERI04G04670)	OgFMO12 (OGLUM04G03920)	OglFMO11 (ORGLA04G0035300)
			OmFMO14 (OMERI04G04680)		
5			OmFMO15 (OMERI05G21010)	OgFMO13 (OGLUM05G24890)	
			OmFMO16 (OMERI05G09400)		
6	ObrFMO10 (OB06G16030)	OpFMO11 (OPUNC06G06240)	OmFMO17 (OMERI06G03520)	OgFMO14 (OGLUM06G06900)	OglFMO12 (ORGLA06G0061000)
		OpFMO12 (OPUNC06G15440)	OmFMO18 (OMERI06G03530)	OgFMO15 (OGLUM06G06910)	OglFMO13 (ORGLA06G0060800)
			OmFMO19 (OMERI06G03580)	OgFMO16 (OGLUM06G14980)	OglFMO14 (ORGLA06G0141900)
			OmFMO20 (OMERI06G07480)	OgFMO17 (OGLUM06G18150)	
			OmFMO21 (OMERI06G07660)		
7	ObrFMO11 (OB07G10720)	OpFMO13 (OPUNC07G00810)	OmFMO22 (OMERI07G09890)	OgFMO18 (OGLUM07G00790)	OglFMO15 (ORGLA07G0007100)
	ObrFMO12 (OB07G10730)	OpFMO14 (OPUNC07G00820)		OgFMO19 (OGLUM07G00830)	OglFMO16 (ORGLA07G0007200)
	ObrFMO13 (OB07G10740)	OpFMO15 (OPUNC07G00840)		OgFMO20 (OGLUM07G11860)	OglFMO17 (ORGLA07G0104200)
	ObrFMO14 (OB07G10800)	OpFMO16 (OPUNC07G11050)			
	ObrFMO15 (OB07G18620)				
8	ObrFMO16 (OB08G20080)				
9	ObrFMO17 (OB09G25280)	OpFMO17 (OPUNC09G17070)		OgFMO21 (OGLUM09G00600)	OglFMO18 (ORGLA09G0148600)
	ObrFMO18 (OB09G25420)			OgFMO22 (OGLUM09G19160)	OglFMO19 (ORGLA09G0148800)
	ObrFMO19 (OB09G25300)			OgFMO23 (OGLUM09G19180)	OglFMO20 (ORGLA09G0149000)
				OgFMO24 (OGLUM09G19210)	
10	ObrFMO20 (OB10G25150)	OpFMO18 (OPUNC10G16400)	OmFMO23 (OMERI10G14540)	OgFMO25 (OGLUM10G04710)	OglFMO21 (ORGLA10G0131800)
	ObrFMO21 (OB10G25220)	OpFMO19 (OPUNC10G16470)	OmFMO24 (OMERI10G14600)	OgFMO26 (OGLUM10G18140)	OglFMO22 (ORGLA10G0132300)
				OgFMO27 (OGLUM10G18190)	
11	ObrFMO22 (OB11G13490)	OpFMO20 (OPUNC11G06230)	OmFMO25 (OMERI11G05170)	OgFMO28 (OGLUM11G05950)	OglFMO23 (ORGLA11G0056800)
	ObrFMO23 (OB11G14940)	OpFMO21 (OPUNC11G14790)	OmFMO26 (OMERI11G08970)	OgFMO29 (OGLUM11G06000)	
12	ObrFMO24 (OB12G14990)	OpFMO22 (OPUNC12G05050)	OmFMO27 (OMERI12G03610)	OgFMO30 (OGLUM12G06000)	OglFMO24 (ORGLA12G0049500)
	ObrFMO25 (OB12G21170)				
**(B) Chromosome**	***O. barthii***	***O. nivara***	***O. indica***	***O. rufipogon***	***O. japonica***
1	ObaFMO1 (OBART01G07810)	OnFMO1 (ONIVA01G10070)	OsIFMO1 (BGIOSGA001676)	OrFMO1 (ORUFI01G08580)	OsJFMO1 (Os01g0224700)
	ObaFMO2 (OBART01G15500)	OnFMO2 (ONIVA01G17610)	OsIFMO2 (BGIOSGA003035)	OrFMO2 (ORUFI01G17290)	OsJFMO2 (Os01g0368000)
	ObaFMO3 (OBART01G24440)	OnFMO3 (ONIVA01G27650)	OsIFMO3 (BGIOSGA003523)	OrFMO3 (ORUFI01G27360)	OsJFMO3 (Os01g0645400)
	ObaFMO4 (OBART01G29910)	OnFMO4 (ONIVA01G34190)	OsIFMO4 (BGIOSGA004101)	OrFMO4 (ORUFI01G33000)	OsJFMO4 (Os01g0732700)
			OsIFMO5 (BGIOSGA004401)		
			OsIFMO6 (BGIOSGA007947)		
2	ObaFMO5 (OBART02G11820)	OnFMO5 (ONIVA02G24200)	OsIFMO7 (BGIOSGA008498)	OrFMO5 (ORUFI02G11880)	OsJFMO5 (Os02g0272200)
	ObaFMO6 (OBART02G21940)			OrFMO6 (ORUFI02G23020)	OsJFMO6 (Os02g0580600)
3	ObaFMO7 (OBART03G04640)	OnFMO6 (ONIVA03G04410)	OsIFMO8 (BGIOSGA011917)	OrFMO7 (ORUFI03G04340)	OsJFMO7 (Os03g0162000)
	ObaFMO8 (OBART03G06010)	OnFMO7 (ONIVA03G06000)	OsIFMO9 (BGIOSGA011989)	OrFMO8 (ORUFI03G05830)	OsJFMO8 (Os03g0182000)
4	ObaFMO9 (OBART04G01320)	OnFMO8 (ONIVA04G01060)	OsIFMO10 (BGIOSGA015543)	OrFMO9 (ORUFI04G01570)	OsJFMO9 (Os04g0128900)
	ObaFMO10 (OBART04G04730)		OsIFMO11 (BGIOSGA015544)	OrFMO10 (ORUFI04G05470)	OsJFMO10 (Os04g0223500)
	ObaFMO11 (OBART04G04740)		OsIFMO12 (BGIOSGA015545)		OsJFMO11 (Os04g0223901)
			OsIFMO13 (BGIOSGA015673)		
5	ObaFMO12 (OBART05G23490)		OsIFMO14 (BGIOSGA017689)	OrFMO11 (ORUFI05G24980)	OsJFMO12 (Os05g0528600)
6	ObaFMO13 (OBART06G06490)	OnFMO9 (ONIVA06G07560)	OsIFMO15 (BGIOSGA021117)	OrFMO12 (ORUFI06G06790)	OsJFMO13 (Os06g0203200)
	ObaFMO14 (OBART06G14000)	OnFMO10 (ONIVA06G07670)	OsIFMO16 (BGIOSGA021291)	OrFMO13 (ORUFI06G14920)	OsJFMO14 (Os06g0203400)
	ObaFMO15 (OBART06G17060)	OnFMO11 (ONIVA06G07680)	OsIFMO17 (BGIOSGA021706)	OrFMO14 (ORUFI06G18060)	OsJFMO15 (Os06g0528700)
		OnFMO12 (ONIVA06G20350)	OsIFMO18 (BGIOSGA021707)		
7	ObaFMO16 (OBART07G00890)	OnFMO13 (ONIVA07G10070)	OsIFMO19 (BGIOSGA024391)	OrFMO15 (ORUFI07G00810)	OsJFMO16 (Os07g0111700)
			OsIFMO20 (BGIOSGA025032)	OrFMO16 (ORUFI07G00820)	OsJFMO17 (Os07g0111900)
			OsIFMO21 (BGIOSGA025033)	OrFMO17 (ORUFI07G12500)	OsJFMO18 (Os07g0112000)
			OsIFMO22 (BGIOSGA025034)		OsJFMO19 (Os07g0112100)
					OsJFMO20 (Os07g0437000)
8		OnFMO14 (ONIVA08G22170)			
9	ObaFMO17 (OBART09G18660)	OnFMO15 (ONIVA09G19750)	OsIFMO23 (BGIOSGA029321)	OrFMO18 (ORUFI09G20190)	OsJFMO21 (Os09g0548400)
	ObaFMO18 (OBART09G18680)	OnFMO16 (ONIVA09G19770)	OsIFMO24 (BGIOSGA029325)	OrFMO19 (ORUFI09G20260)	OsJFMO22 (Os09g0548700)
	ObaFMO19 (OBART09G18710)	OnFMO17 (ONIVA09G19830)	OsIFMO25 (BGIOSGA029326)		OsJFMO23 (Os09g0549300)
		OnFMO18 (ONIVA10G13930)			
10	ObaFMO20 (OBART10G18040)	OnFMO19 (ONIVA10G14730)	OsIFMO26 (BGIOSGA031436)	OrFMO20 (ORUFI10G19230)	OsJFMO24 (Os10g0553800)
	ObaFMO21 (OBART10G18090)	OnFMO20 (ONIVA10G14740)	OsIFMO27 (BGIOSGA031433)	OrFMO21 (ORUFI10G19280)	OsJFMO25 (Os10g0554300)
		OnFMO21 (ONIVA10G20590)			
		OnFMO22 (ONIVA10G20640)			
11	ObaFMO22 (OBART11G06190)	OnFMO23 (ONIVA11G06260)	OsIFMO28 (BGIOSGA034995)	OrFMO22 (ORUFI11G06350)	OsJFMO26 (Os11g0207700)
	ObaFMO23 (OBART11G06210)			OrFMO23 (ORUFI11G06380)	OsJFMO27 (Os11g0207900)
12	ObaFMO24 (OBART12G05080)	OnFMO24 (ONIVA12G04710)	OsIFMO29 (BGIOSGA036502)	OrFMO24 (ORUFI12G05850)	OsJFMO28 (Os12g0189500)
	ObaFMO25 (OBART12G13520)				

**Table 2:** **Distribution of FMO genes in 10 genomes of rice.** The chromosome-wise distribution of the identified FMO family members in the genomes of ten *Oryza* species, arranged according to their evolutionary history. *O. brachyantha* is the most distant and *O. sativa* subp. *japonica* is the most recent among these species. Each row contains list of genes that are orthologs of each other in different *Oryza* species. The blank space denotes the absence of the corresponding ortholog in the respective species. * Indicates the presence of an ortholog that belongs to some different chromosome in a particular row.

Chromosome	*O. brachyantha*	*O. punctata*	*O. meridionalis*	*O. glumaepatula*	*O. glaberrima*	*O. barthii*	*O. nivara*	*O. indica*	*O. rufipogon*	*O. japonica*
1	ObrFMO1	OpFMO1	OmFMO1	OgFMO1	OglFMO1	ObaFMO1	OnFMO1	OsIFMO2	OrFMO1	OsJFMO1
	ObrFMO2	OpFMO2		OgFMO2						
		OpFMO3	OmFMO3	OgFMO4	OglFMO2	ObaFMO2		OsIFMO3	OrFMO2	OsJFMO2
	ObrFMO3	OpFMO4	OmFMO4	OgFMO5	OglFMO3	ObaFMO3	OnFMO3	OsIFMO4	OrFMO3	OsJFMO3
	ObrFMO4	OpFMO5	OmFMO5	OgFMO6	OglFMO4	ObaFMO4	OnFMO4	OsIFMO5	OrFMO4	OsJFMO4
2			OmFMO8		OglFMO5	ObaFMO5		OsIFMO6	OrFMO5	OsJFMO5
	ObrFMO5	OpFMO6	OmFMO6, OmFMO16 *	OgFMO7	OglFMO6	ObaFMO6	OnFMO5	OsIFMO7	OrFMO6	OsJFMO6
3	ObrFMO6	OpFMO7	OmFMO9	OgFMO8	OglFMO7	ObaFMO7	OnFMO6	OsIFMO8	OrFMO7	OsJFMO7
	ObrFMO7	OpFMO8	OmFMO10	OgFMO9	OglFMO8	ObaFMO8	OnFMO7	OsIFMO9	OrFMO8	OsJFMO8
4	ObrFMO8	OpFMO9	OmFMO11, OmFMO2 *	OgFMO21 *, OgFMO3	OglFMO9	ObaFMO9	OnFMO8, OnFMO2 *	OsIFMO1, OsIFMO13	OrFMO9	OsJFMO9
	ObrFMO9	OpFMO10	OmFMO13	OgFMO11	OglFMO10	ObaFMO10	OnFMO19 *	OsIFMO11, OsIFMO12		OsJFMO10
			OmFMO12 OmFMO14	OgFMO10, OgFMO12	OglFMO11	ObaFMO11	OnFMO20 *	OsIFMO10	OrFMO10	OsJFMO11
			OmFMO15	OgFMO13		ObaFMO12	OnFMO14 *	OsIFMO14	OrFMO11	OsJFMO12
5	ObrFMO10		OmFMO20, OmFMO21	OgFMO14	OglFMO12	ObaFMO13	OnFMO9	OsIFMO18		OsJFMO13
6							OnFMO10			
		OpFMO11		OgFMO15	OglFMO13		OnFMO11	OsIFMO17	OrFMO12	OsJFMO14
		OpFMO12		OgFMO17	OglFMO14	ObaFMO15	OnFMO12	OsIFMO15	OrFMO14	OsJFMO15
			OmFMO26 *	OgFMO25, OgFMO16 *		ObaFMO14		OsIFMO16, OsIFMO22	OrFMO13	OsJFMO18 *
7	ObrFMO11, ObrFMO12, ObrFMO13	OpFMO13		OgFMO18		ObaFMO16	OnFMO18 *	OsIFMO20	OrFMO15	OsJFMO16, OsJFMO17
	ObrFMO14, ObrFMO22, ObrFMO16 *	OpFMO14, OpFMO15	OmFMO7 *	OgFMO19	OglFMO15, OglFMO16			OsIFMO21	OrFMO16	OsJFMO19
	ObrFMO15	OpFMO16	OmFMO22	OgFMO20	OglFMO17		OnFMO13	OsIFMO19	OrFMO17	OsJFMO20
9	ObrFMO17	OpFMO17	OmFMO17 *	OgFMO22	OglFMO18	ObaFMO17	OnFMO15	OsIFMO25	OrFMO18	OsJFMO21
	ObrFMO19		OmFMO18 *	OgFMO23	OglFMO19	ObaFMO18	OnFMO16	OsIFMO24	-	OsJFMO22
	ObrFMO18		OmFMO19 *	OgFMO24	OglFMO20	ObaFMO19	OnFMO17	OsIFMO23	OrFMO19	OsJFMO23
10	ObrFMO20	OpFMO18	OmFMO23	OgFMO26	OglFMO21	ObaFMO20	OnFMO21	OsIFMO26	OrFMO20	OsJFMO24
	ObrFMO21	OpFMO19	OmFMO24	OgFMO27	OglFMO22	ObaFMO21	OnFMO22	OsIFMO27	OrFMO21	OsJFMO25
11		OpFMO20	OmFMO25	OgFMO28	OglFMO23	ObaFMO22			OrFMO22, OrFMO23	OsJFMO26
	ObrFMO23	OpFMO21		OgFMO29		ObaFMO23	OnFMO23	OsIFMO28		OsJFMO27
12	ObrFMO24	OpFMO22	OmFMO27	OgFMO30	OglFMO24	ObaFMO24	OnFMO24	OsIFMO29	OrFMO24	OsJFMO28
	ObrFMO25					ObaFMO25				-

## Data Availability

The datasets supporting the conclusions of this article are included within the article and its additional files. The sequence data for all the *Oryza* species were obtained from Gramene data resource (https://gramene.org (accessed on 12 July 2022). For *O. sativa* subsp. *japonica*, the sequences were also retrieved from the RGAP (http://rice.plantbiology.msu.edu/ (accessed on 15 September 2021)).

## Supplementary Materials

The following supporting information can be downloaded at: https://www.mdpi.com/article/10.3390/ijms24044190/s1.
